# The effect of yoga on the delivery and neonatal outcomes in nulliparous pregnant women in Iran: a clinical trial study

**DOI:** 10.1186/s12884-021-03794-6

**Published:** 2021-05-03

**Authors:** Leili Yekefallah, Peyman Namdar, Leila Dehghankar, Fereshteh Golestaneh, Soghra Taheri, Frahnaz Mohammadkhaniha

**Affiliations:** 1 Department of Nursing, Metabolic Diseases Research Center, Research Institute for Prevention of Non-Communicable Diseases, School of Nursing & Midwifery, Qazvin University of Medical Sciences, Qazvin, Iran; 2 Department of Emergency Medicine, Metabolic Disease Research Center, Qazvin University of Medical Sciences, Qazvin, Iran; 3 Bsc in Midwifery, Pregnancy Yoga Instructor, Shahid Beheshti University of Medical Sciences, Tehran, Iran; 4 Clinical Research Development Unit, Kowsar Hospital, Qazvin University of Medical Science, Qazvin, Iran

**Keywords:** Yoga, Pregnancy outcome, Neonatal

## Abstract

**Background:**

Yoga can reduce the risk of preterm delivery, cesarean section (CS), and fetal death. The aim of the present study was to investigate the effects of Yoga on pregnancy, delivery, and neonatal outcomes.

**Methods:**

This was a clinical trial study and using the random sampling without replacement 70 pregnant women entered Hatha Yoga and control groups according to the color of the ball they took from a bag containing two balls (blue or red). The data collection tool was a questionnaire pregnancy, delivery, and neonatal outcomes. The intervention in this study included pregnancy Hatha Yoga exercises that first session of pregnancy Yoga started from the 26th week and samples attended the last session in the 37th week. They exercised Yoga twice a week (each session lasting 75 min) in a Yoga specialized sports club. The control group received the routine prenatal care that all pregnant women receive.

**Results:**

The results showed that yoga reduced the induction of labor, the episiotomy rupture, duration of labor, also had a significant effect on normal birth weight and delivery at the appropriate gestational age. There were significant differences between the first and second Apgar scores of the infants.

**Conclusion:**

The results of the present study showed that Yoga can improve the outcomes of pregnancy and childbirth. They can be used as part of the care protocol along with childbirth preparation classes to reduce the complications of pregnancy and childbirth.

**Trial registration:**

IRCT20180623040197N2 (2019-02-11).

## Background

The pregnancy stage has a profound effect on the life of every woman and is associated with physical and psychological changes. During this period, attention to the health of the fetus is of particular importance [1, 2]. The maternal mortality rate due to elective Cesarean Section (CS) is 2 to 3 times higher than that of natural delivery [3]. The high rates of CS increase maternal (such as uterine infections, postpartum hemorrhage, and anesthesia) and neonatal (such as increased length of hospital stay in the neonatal intensive care unit, respiratory diseases, jaundice, neonatal infection, low Apgar score, low birth weight, and neonatal mortality) complications [3, 4].

Prenatal care and type of delivery for nulliparous pregnant women have a great impact on their experience of pregnancy and delivery and can also determine the type of their next delivery [3]. In the health system transformation plan, the promotion of natural childbirth is pursued with the general goal of improving the health indicators of mothers and infants by reducing the CS rate [5]. However, one of the complications of natural childbirth is episiotomy which is much more common in Iran than in other countries [6]. Episiotomy has such side effects as delayed wound healing, increased risk of infection, fatigue, insomnia, difficulty in sitting and holding the infant, decreased sexual function, and impaired emotional relationships between the mother and infant [6]. In their study, Firuzbakht et al. reported the effect of childbirth preparation classes and exercises on reducing the rates of episiotomy and emergency CS [7].

There is a problem of reduced physical activity during pregnancy so that 60% of women become sedentary during this period [8]. On the other hand, due to their lack of awareness about the benefits of exercise during pregnancy, women are reluctant to exercise [9]. Exercise during pregnancy has a high priority in terms of health and care due to its benefits for the mother and fetus [10].

Exercise during pregnancy is one of the best ways to reduce pregnancy complications such as insomnia, feeling tired, excessive weight gain of mother, back and low back pain, pelvic pain, constipation, urinary incontinence, hypertension, gestational diabetes, depression, and anxiety. In addition, exercise increases individuals’ ability to adapt to activities related to infant care [8].

There are different types of physical activities during pregnancy, such as yoga, pregnancy gymnastics, Pilates, and Kegel exercises. One of the physical activities that pregnant women tend to do is pregnancy yoga [11]. Numerous studies have shown that yoga improves the outcomes of low-risk and high-risk pregnancies [12]. Yoga interventions contribute to both physical and psychological health. They improve muscle strength, memory, and sleep quality and reduce pain and depression [13]. In ancient Indian science, yoga has been described as a lifestyle including changes in mental attitude and diet besides the practice of certain techniques [9]. Yoga is an ideal way to spend the pregnancy period [14].

Yoga during pregnancy allows pregnant women to make connections with their mind, body, soul, and fetus [1, 15]. Field et al. concluded that yoga exercises during pregnancy reduced anxiety, depression, and the back and pelvic pains and led to delivery at an appropriate gestational age and the normal birth weight of infants [16]. In their study, Jahdi et al. examined the effect of yoga on the severity of labor pain and the outcomes of labor and reported the significant effect of yoga on reducing CS and induction of labor [17]. Furthermore, Ostrovesky stated that yoga during pregnancy can reduce the rate of CS and labor pain [18].

Yoga is usually a combination of mental exercises, meditation, various types of deep breathing, stretching, and relaxation [1, 18]. Meditation is a specialized exercise which provides deep relaxation to calm the body and focus the mind [19]. The benefits of yoga include a better physical growth, stronger and more flexible muscles and joints, and the reduced risks of preterm delivery, hypertension due to pregnancy, and intrauterine growth restriction [13]. Yoga techniques are non-invasive and have fewer side effects [20] and so far no study has reported the side effects or negative effects of yoga exercises on the physiological or psychological outcomes of pregnant mothers [1, 14].

Yoga exercises during pregnancy can reduce the risks of preterm delivery, intrauterine growth restriction, preeclampsia (high blood pressure during pregnancy), emergency CS, and fetal death [21, 22]. In their study, entitled “the effectiveness of yoga on pregnancy outcomes (pregnancy age and infant weight)”, Narendran et al. reported that the abnormal birth weight, preterm delivery, intrauterine growth restriction, and gestational hypertension were significantly lower in the yoga group than in the control group [23]. In their study, Rakhshani et al. examined the effect of yoga on the pregnancy outcomes of 68 women with high-risk pregnancy and showed that pregnancy yoga reduced the outcomes of gestational diabetes, hypertension, preeclampsia, and intrauterine growth restriction, and preterm delivery, low birth weight of the infant, and low Apgar score [20].

Given that pregnancy yoga is a non-invasive technique with few side effects, that no study has been found on the side effects or negative effects of yoga exercises on pregnant women, and that yoga can be performed by most pregnant women, also due to the contradictory results in some studies and few studies regarding the effectiveness of this type of yoga in nulliparous women the authors decided to conduct a study to investigate the effects of yoga on pregnancy, delivery, and neonatal outcomes in Qazvin, Iran.

## Methods

This study was a clinical trial study in which the study population included all pregnant mothers who referred to the clinic of Kowsar Hospital. The inclusion criteria included the informed consent of the samples to participate in the study, the age group of 18 to 35 years, normal pregnancy according to the written diagnosis of a gynecologist, first and singleton pregnancy, the ability to read and write, normal body mass index (between 19.8 to 26), the gestational age of 26 to 28 weeks, no physical or mental disability according to the doctor’s written diagnosis, enough time to participate in yoga sessions, the ability to do the yoga exercises (from the patient’s point of view), and no musculoskeletal disorders. The exclusion criteria included being infected with a specific disease, pregnancy complications (gestational diabetes and preeclampsia), experience of attending a yoga class or regular exercise (Pilates, Tai Chi), abnormal fetus or IUGR, elective CS, absence of more than two sessions, and the subjects’ lack of cooperation during the yoga exercises according to the yoga instructor.

According to an article by Jahdi et al., the mean scores of pain intensity in the yoga and control groups were reported as 2.0 ± 6.9 and 3.5 ± 1.4, respectively. Therefore, by taking account of the confidence interval of 95% (α = 0.05) and the test power of 80% (β = 0.2), the sample size was calculated using the following eq. [17].

$$ n=\frac{{\left({z}_{1-\frac{\alpha }{2}}+{z}_{1-\beta}\right)}^2\left({\sigma}_1^2+{\sigma}_2^2\right)}{{\left({\mu}_2-{\mu}_1\right)}^2}=\frac{{\left(1.86+0.84\right)}^2\left({0.9}^2+{1.4}^2\right)}{0.9^2}=32 $$

Considering a 10% drop, the final sample size was increased to 70 women.

For sampling, eligible nulliparous pregnant women entered the study using the convenience sampling method. Then, for randomize assignment, women selected were assigned to odd days to control group and women selected in even days to intervention group. Randomization ratio considered 1:1 at first, then due to high sample drop out, in two groups, sampling process continued until the final sample size in each group obtained.

After completing the sampling, the research objectives were explained to all the pregnant women in two sessions and if they were satisfied and completed the relevant form, the demographic information questionnaires would be completed for them. Moreover, their addresses and phone numbers were taken to inform them of the start of the yoga sessions. The intervention in this study included pregnancy Hatha yoga exercises taught by a trained yoga instructor with an international certification and a 15-year history of teaching yoga to pregnant women. The participants in the intervention group started the first session of pregnancy yoga from the 26th to 28th weeks of pregnancy and attended the last session in the 37th week of pregnancy. They exercised yoga twice a week (each session lasting 75 min) in a yoga specialized sports club. The control group received the routine prenatal care that all pregnant women receive. In the intervention group, the yoga exercises included physical movements for 50 min (such as triangle exercises, angled exercises, archery position, pelvic rotation in a standing position, joyful jumps, rotation and release, pelvic floor stretches, strengthening the pelvic floor muscles, gentle perineal traction, upward pelvic rotation, pink panther steps, hip release, knee rotation, active traction in the kneeling position, knee movements, hip and knee cycles, and knee sitting), meditation for 15 min (deep relaxation with focus on breathing and self-awareness), breathing exercises in between, and relaxation for 10 min [24].

The data collection tool was a questionnaire consisted of two parts:

A) A demographic characteristics questionnaire (about age, education, occupation, underlying disease, height, weight, body mass index, and residence) which was completed before the intervention.
B) Information about the pregnancy, childbirth (the type of delivery, use of local or general anesthesia, induction of labor, duration of labor, gestational age at delivery, birth weight, premature rupture of membranes, preterm labor, intrauterine growth restriction, preeclampsia, episiotomy, and rupture grade), and neonatal outcomes (through the Apgar score at the first and 5 minutes after birth including measuring the infant’s heart rate, respiratory effort, muscle stiffness, irritability, appearance, and skin color). A score above seven indicated the proper condition of the infant. The duration of labor was from the onset of pain until the birth of the infant and the separation of the placenta [19, 21].

It should be noted that to compare the two groups, the questionnaire of the pregnancy and delivery outcomes was completed for all the participants using medical records.

After collecting the data, descriptive statistics including frequency, relative frequency, mean, and standard deviation were used to describe the samples. The Kolmogorov–Smirnov test was used to evaluate the normality of the variables. Then, due to the non-normality of the data, the Mann-Whitney test was used to compare the means of the quantitative variables in both groups. Besides, the chi-square test was used to examine the qualitative variables in both groups. The data were analyzed using the SPSS software (version 24) and the significance level was considered 0.05.

To carry out the present study, the written approval of the Ethics Committee of Qazvin University of Medical Sciences was obtained (code: 184.IR.QUMS.REC.1397). This study was registered in the Iranian Registry of Clinical Trials (code: IRCT20180623040197N2).

## Results

The results of this study showed that the mean and standard deviation of the age of the participants in the yoga and control groups were 27.45 ± 4.50 and 26.82 ± 4.38 years, respectively. Moreover, the mean and standard deviation of BMI in the yoga and control groups were 23.87 ± 2.30 and 23.47 ± 2.21, respectively. Most of the participants in the yoga group (34.3%) and the control group (37.1%) had a bachelor’s degree. Besides, 71.4% of the participants in the yoga group and 45.7% in the control group were housekeepers. There was no significant difference between the demographic characteristics of the pregnant women in the yoga and control groups and both groups were homogeneous (Table 1).

**Table 1:** Demographic characteristics of mothers in the intervention and control groups

***Quantitative variables***		**Yoga group (35)**	**Control group (35)**	**Test results**	**df**	**p**
		**N (%)**	**N (%)**			
*Education*	Elementary	3 (8.6%)	9 (25.7%)	χ 2 = 6.31	3	*p* = 0.097
	Diploma	6 (17.1%)	7 (20.0%)			
	Higher than diploma	12 (34.3%)	13 (37.1%)			
	Undergraduate	14 (40.0%)	6 (17.1%)			
*Job*	Employee	6 (17.1%)	8 (22.9%)	χ 2 = 5.52	2	*p* = 0.063
	Self-employed	4 (11.4%)	11 (31.4%)			
	Housekeeper	25 (71.4%)	16 (45.7%)			
*Residence*	Qazvin	25 (71.4%)	20 (57.1%)	χ 2 = 6.31	2	*p* = 0.035
	County around	8 (22.9%)	10 (28.6%)			
	Other	2 (5.7%)	5 (14.3%)			
***Quantitative variables***		**Mean (SD)**	**Mean (SD)**	**t-test**	**df**	**p**
*Age*		27.45 ± 4.50	26.82 ± 4.38	t = 0.592	68	*p* = 0.590
*BMI*		23.87 ± 2.30	23.47 ± 2.21	t = 0.740	68	*p* = 0.570

The results showed that yoga exercises had a significant effect on the rate of induction of labor in pregnant women in the yoga group (*p* = 0.02, χ 2 = 5.41) so that 51.7% of the women in the yoga group and 82.6% of the women in the control group could have natural childbirth using induction of labor. Pregnancy yoga exercises had a significant effect on the variable of preterm delivery outcome in the yoga group (*p* = 0.03, χ 2 = 4.24) so that 0% of the subjects in the yoga group and 11.4% in the control group had preterm labor and delivery before the 37th week of pregnancy. The results showed that 82.9% of the women in the yoga group and 65.7% in the control group had a natural delivery and the rest underwent emergency CS for various reasons. Hence, there was a significant statistical difference between the two groups (*p* = 0.044, χ 2 = 4.05). In addition, 77.1% of the pregnant women in the yoga group and 65.7% in the control group performed an episiotomy during natural delivery which was not statistically significant (*p* = 0.29, χ 2 = 1.12). However, there was a significant difference in the rupture grade of episiotomy (*p* < 0.0001, χ 2 = 29.08) so that 55.2% of the women in the yoga group did not have a rupture and 44.8% had a first-grade rupture, while, in the control group, 43.5% had a first-grade rupture, 47.8% had a second-grade rupture, and 8.7% had a third-grade rupture (Table 2).

**Table 2:** The outcomes of pregnancy and delivery in the yoga and control groups

Outcomes		Yoga group (35)	Control group (35)	test	***p***
**Natural delivery**	Yes	29 (82.9%)	23 (65.7%)	χ 2 = 2.69	*p* = 0.101
	No	6 (17.1%)	12 (34.3%)		
**Cesarean section**	Yes	8 (22.9%)	16 (45.7%)	χ 2 = 4.05	*p* = 0.044
	No	27 (77.1%)	19 (54.3%)		
**Use of local anesthesia**	Yes	28 (80.0%)	13 (37.1%)	χ 2 = 2.52	*p* = 0.112
	No	7 (20.0%)	22 (62.9%)		
**Use of general anesthesia**	Yes	5 (14.3%)	7 (20.0%)	χ 2 = 0.40	*p* = 0.52
	No	30 (85.7%)	28 (80.0%)		
**Induction of labor**	Yes	15 (51/7%)	19 (82/6%)	χ 2 = 5/41	*p* = 0.02
	No	14 (48/3%)	4 (17/4%)		
**Premature rupture of the membranes**	Yes	9 (25.7%)	12 (34.3%)	χ 2 = 0.612	*p* = 0.43
	No	26 (74.3%)	23 (65.7%)		
**Preterm delivery**	Yes	0 (0%)	4 (11.4%)	χ 2 = 4.24	*p* = 0.039
	No	35 (100.0%)	31 (88.6%)		
**Episiotomy**	Yes	27 (77.1%)	23 (65.7%)	χ 2 = 1.12	*p* = 0.29
	No	8 (22.9%)	12 (34.3%)		
**Episiotomy grade**	0	16 (55.2%)	0 (0.0%)	χ 2 = 29.08	*p* < 0.0001
	1	13 (44.8%)	10 (43.5%)		
	2	0 (0.0%)	11 (47.8%)		
	3	0 (0.0%)	2 (8.7%)		

The results showed that yoga exercises had a significant effect on the infant birth weight in nulliparous women (*p* = 0.001, U = 330.000) and the mean birth weight of the infants in the yoga group was higher than that of the control group so that the mean and standard deviation of the infant birth weight in the yoga and control groups were 3287.28 ± 369.54 and 2974.85 ± 301.26, respectively. The mean and standard deviation of the gestational age at delivery were 38.98 ± 0.99 in the yoga group and 37.32 ± 1.78 in the control group. Subsequently, the Mann-Whitney test showed a significant difference between the two groups (*p* < 0.0001, U = 259.000) (Table 3).

**Table 3:** The pregnancy and neonatal outcomes in the yoga and control groups

Outcomes	Yoga group (35)	Control group (35)	Test	***p***
	Mean (SD)	Mean (SD)		
**Birth weight**	3287.28 ± 369.54	2974.85 ± 301.26	Mann-Whitney U = 330.000	*p* = 0.001
**Gestational age**	38.98 ± 0.99	37.32 ± 1.78	Mann-Whitney U = 259.000	*p* < 0.0001
**Labor duration**	3.1 ± 0.96	4.91 ± 1.53	Mann-Whitney U = 112.000	*p* < 0.0001
**First minute Apgar** **score**	8.94 ± 0.23	8.54 ± 0.98	Mann-Whitney U = 469.500	*p* = 0.010
**Fifth minute Apgar** **score**	9.94 ± 0.23	9.28 ± 0.66	Mann-Whitney U = 260.500	*p* < 0.0001

The results showed that the mean duration of labor from the onset of pain until natural labor was 3.1 ± 0.96 in the yoga group and 4.91 ± 1.53 in the control group, which was statistically significant (*p* < 0.0001, U = 112.000). Furthermore, yoga exercises had a significant effect on the Apgar score in the first to fifth minutes of labor in nulliparous women. There were significant differences between the first Apgar scores of the infants in the control group and the yoga group (*p* = 0.010, U = 469.500) and between the second Apgar scores of the infants in the control group and the yoga group (*p* < 0.0001, U = 260.500) (Table 3).

## Discussion

This study aimed to investigate the effect of yoga on neonatal and pregnancy outcomes in Qazvin. The results showed that yoga exercises had a significant impact on the induction of natural labor in pregnant women in the yoga group so that most nulliparous women in the yoga group went to the hospital with normal pain and had a short-term natural delivery (which was statistically significant). In contrast, in the control group, most pregnant women were able to have a natural delivery using induction of labor and the duration of labor (from the onset of pain to the separation of the fetus) was longer. The study of Mohyadin et al. showed that yoga therapy caused fewer women to use natural labor induction, whereas, in the control group, more women used labor induction. Furthermore, there was a shorter duration of labor in the yoga group, which was statistically significant [25].

In the study of Jahdi et al., fewer mothers in the intervention group needed induction of labor compared to those of the control group. Furthermore, the intervention group had fewer numbers of CS than the control group and experienced a shorter duration of the second and third stages of labor [17]. In the study of Chuntharapat, in the intervention group, the first stage of the labor was shorter and the delivery duration was significantly reduced [19]. In the study of Khavandizadeh et al., childbirth preparation classes led to a shortening of the duration of labor [26]. Combined exercise can have a beneficial effect on changes and pain during pregnancy, including the beneficial effects of help to prepare the body to withstand the pressure of childbirth, help reduce back pain, help the body to carry extra weight more effectively during pregnancy, improved blood circulation, lowering blood pressure and heart rate. Yoga exercises can relieve muscle tension that may develop during pregnancy and increase the flexibility, muscle strength, endurance, and energy needed for labor, making it easier and shorter [15, 27].

Exercise and yoga during pregnancy do not increase preterm labor and evidence has shown that regular exercise and pregnancy yoga (with moderate-to-heavy intensity) during pregnancy reduce preterm labor [25, 28]. The results of the study of Rakhshani et al. showed that preterm delivery and intrauterine growth restriction were significantly reduced in the intervention group [20]. In their study, Field et al. reported that preterm delivery was lower in the yoga and massage therapy groups than in the control group, which was statistically significant [16]. In their study on the effectiveness of yoga on pregnancy outcomes, Narendran et al. stated that preterm delivery and intrauterine growth restriction were significantly lower in the yoga group than in the control group [23].

The results showed that the pregnant women in the yoga group had more natural delivery than the control group and only a few of them underwent emergency CS for various reasons, showing a statistically significant difference between the two groups. The study of Mohyadin et al. showed that there were more natural deliveries than emergency CS in the yoga group, though it was not statistically significant [25]. In the study of Rakhshani et al., no significant difference was observed between the two groups regarding the emergency CS and premature rupture of the membranes [20]. Ostrovesky stated that yoga during pregnancy could reduce the rate of CS and labor pain [18]. The study of Khavandizadeh et al. also showed that childbirth preparation classes led to a reduction in CS [26]. Many yoga exercises are stretching exercises which are expected to increase the tensile strength and flexibility of the vaginal and perineal muscles. Hence, yoga exercises can possibly facilitate vaginal delivery and help shorten the duration of delivery [25]. The differences in the results of the mentioned studies could be due to the differences in the nutrition of mothers, the volume and intensity of exercise, hereditary backgrounds, and the socio-economic status of mothers. CS is one of the major surgeries that is sometimes associated with very dangerous and deadly complications. Therefore, most countries, as well as Iran, try to reduce the number of CS by performing interventions. Yoga exercises have become one of the effective interventions to reduce the number of CS.

The pregnant women in the yoga and control groups performed an episiotomy during natural delivery, which was not statistically significant. However, there was a significant difference in the episiotomy rupture grade so that in the yoga group women had the rupture grades of one and zero, whereas in the control group, they had the rupture grades of one, two, and three. Episiotomy has such side effects as delayed wound healing, increased risk of infection, fatigue, insomnia, difficulty in sitting and holding the infant, decreased sexual function, and impaired emotional relationships between the mother and infant [6]. In their study, Firuzbakht et al. showed the effect of childbirth preparation classes and exercises on reducing the rate of episiotomy [7]. The study of Sharifizad et al. demonstrated that complaints of episiotomy pain and postpartum hemorrhage were significantly lower in the natural delivery group than in the CS group [29].

The results showed that yoga exercises had a significant effect on the birth weight of nulliparous women’s infants and the mean birth weight of the infants in the yoga group was higher than that of the control group. Field et al. showed that the infants’ weight was higher in the yoga and massage therapy groups than that of the control group, which was statistically significant [16]. In their study on the effectiveness of yoga on pregnancy outcomes (gestational age and infant weight), Narendran et al. acknowledged that the birth rate of infants with an abnormal weight was significantly lower in the yoga group than in the control group [23]. It should be noted that one of the factors affecting the birth weight of the infant is the regular repetition of yoga exercises during pregnancy, which is one of the reasons for the difference in the results of different studies.

The results of the present study showed that the mean and standard deviation of gestational age at delivery in the yoga group were higher than those of the control group, showing a significant difference between the two groups. In the study of Rakhshani et al. [20] and Field et al. [16], delivery at a low gestational age was less in the intervention group, which was statistically significant [20].

Yoga exercises had a significant effect on the Apgar score in the first to fifth minutes of labor in nulliparous women. There were significant differences between the first Apgar scores of the infants in the control group and the yoga group and between the second Apgar scores of the infants in the control group and the yoga group. In the study of Rakhshani et al., the number of infants with a low Apgar score and the rate of delivery at a low gestational age were lower in the intervention group than in the control group [20]. Gehan et al. also showed in their research that exercise during pregnancy in nulliparous women led to a significant statistical difference between the intervention and control groups regarding the Apgar score in the first and fifth minutes [30]. In the study of Mohyadin et al. [25], Jahdi [17], and Chuntharapat, no significant difference was observed between the yoga and control groups regarding the infants’ Apgar scores in the first and fifth minutes [19]. The reasons for the differences between these studies were the differences in the nature, type, intensity, and frequency of yoga exercises in pregnant women during the pregnancy period.

The limitations of this study, which were not under the researchers’ control included the small sample size, unrecognizable emotional and mental conditions unexpressed by the participants, the stress and anxiety of the pregnant women during childbirth, and environmental factors (such as disturbing noises which were minimized in this study). It is suggested that further research be done with larger sample sizes, other factors affecting the delivery and neonatal outcomes be taken into account, and other scales be used.

## Conclusion

The results of the present study showed that yoga exercises could lead to a normal birth weight and improve the infant’s Apgar score and reduce emergency CS, labor duration, induction of labor, and preterm labor. Therefore, they can be used as part of the care protocol along with childbirth preparation classes to reduce the complications of pregnancy and delivery. Therefore, yoga exercises can be part of the usual midwifery nursing care. Since these exercises are low-cost and uncomplicated, they can be used by nurses, midwives, and gynecologists for willing nulliparous pregnant women.

## Practice points

- When women are at risk for preterm labor based on the presence of risk factors, give advice on modifiable risk factors.
- Explain options with a woman who is nearing prolong pregnancy, including induction of labor.
- Midwifery-led care is associated with the best outcomes for low-risk deliveries
- Assess the baby’s Apgar regularly
- For prolonged second stage of labor, consider the options of cesarean section versus assisted vaginal birth
- Avoid routine episiotomy
- Avoid routine preterm labor

## Research agenda

- The effect of reducing the rate of CS is introduced when yoga exercises are performed during pregnancy.
- Determining the extent of adverse neonatal and delivery
- Definition of failed induction, compared with shorter time intervals and different access to Cesarean delivery in yoga
- Further empirical research is needed to reach a consensus on the starting criteria (both in terms of definition and support).

## Data Availability

The data that support the findings of this study are available from [Leila Dehghankar] but restrictions apply to the availability of this data, which were used under license for the current study, and so are not publicly available. Data are however available from the authors upon reasonable request and with permission of [Leila Dehghankar].

## Abbreviations

CS: Cesarean section
